# Application of alternative models to identify QTL for growth traits in an F_2 _Duroc x Pietrain pig resource population

**DOI:** 10.1186/1471-2156-11-97

**Published:** 2010-11-01

**Authors:** Igseo Choi, Juan P Steibel, Ronald O Bates, Nancy E Raney, Janice M Rumph, Catherine W Ernst

**Affiliations:** 1 Department of Animal Science, Michigan State University, East Lansing, Michigan, USA

## Abstract

**Background:**

A variety of analysis approaches have been applied to detect quantitative trait loci (QTL) in experimental populations. The initial genome scan of our Duroc x Pietrain F_2 _resource population included 510 F_2 _animals genotyped with 124 microsatellite markers and analyzed using a line-cross model. For the second scan, 20 additional markers on 9 chromosomes were genotyped for 954 F_2 _animals and 20 markers used in the first scan were genotyped for 444 additional F_2 _animals. Three least-squares Mendelian models for QTL analysis were applied for the second scan: a line-cross model, a half-sib model, and a combined line-cross and half-sib model.

**Results:**

In total, 26 QTL using the line-cross model, 12 QTL using the half-sib model and 3 additional QTL using the combined line-cross and half-sib model were detected for growth traits with a 5% false discovery rate (FDR) significance level. In the line-cross analysis, highly significant QTL for fat deposition at 10-, 13-, 16-, 19-, and 22-wk of age were detected on SSC6. In the half-sib analysis, a QTL for loin muscle area at 19-wk of age was detected on SSC7 and QTL for 10th-rib backfat at 19- and 22-wk of age were detected on SSC15.

**Conclusions:**

Additional markers and animals contributed to reduce the confidence intervals and increase the test statistics for QTL detection. Different models allowed detection of new QTL which indicated differing frequencies for alternative alleles in parental breeds.

## Background

A variety of analysis approaches have been applied to detect quantitative trait loci (QTL) in experimental populations. For an F_2 _population design, a line-cross model is most commonly used to detect QTL segregating between divergent lines. This model assumes the founder lines are fixed for alternative QTL alleles [1] and under such assumption is the most powerful [2]. However, the QTL effects under the line-cross model can be biased downwards since not all QTL alleles are completely fixed, especially in domestic animals [3]. In addition, introgression of QTL detected using the line-cross model is difficult since genetic improvement in the pig breeding industry has been achieved largely by within breed selection [4]. To identify QTL segregating within parental breeds, a half-sib model that does not assume fixation of QTL alleles in the founder lines was introduced by Knott et al. [5]. A general model that accounts for between and within line segregation has been proposed [3], but it is computationally prohibitive to implement in many populations. Kim et al. [6] subsequently developed a combined model which accounts for both line effects and half-sib family effects.

Along with appropriate statistical methods for QTL mapping, marker density and sample size are also determining factors for estimating QTL locations and effects with accuracy and precision. Although increasing marker density is becoming routine for high resolution mapping [7], a two-step strategy of adding markers and animal genotypes into previously identified QTL regions is efficient and cost effective.

We have previously reported results for a whole genome scan of our Duroc x Pietrain F_2 _population using a line-cross analysis [8,9]. Both the Duroc and Pietrain breeds are used in commercial pig production and they exhibit variation in growth phenotypes [8]. The objective of this study was to detect new QTL for growth traits using three different models, and to refine previously identified QTL regions with addition of new markers and additional F_2 _animals.

## Results

**Figure 1:**
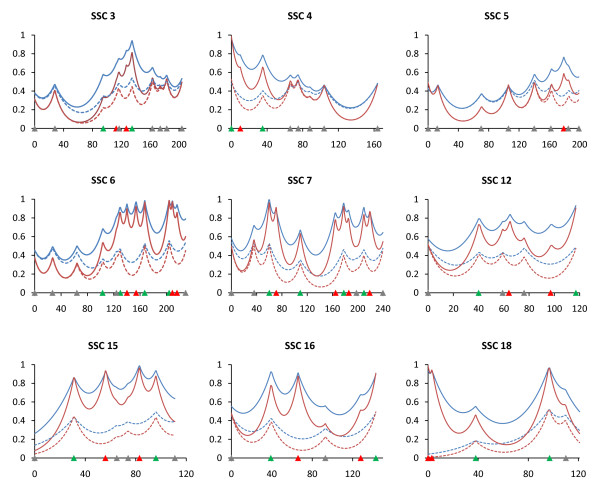
**Effect of additional markers and animals on information content**. Information content was determined for both the first QTL scan (dotted lines) and the second scan (solid lines) which include additional animals and markers. Blue lines indicate additive effects and red lines indicate dominance effects. Marker positions are shown as triangles on the X-axis (gray, markers used for both QTL scans and genotyped only in 510 animals; green, markers used for both QTL scans and genotyped in all animals; red, markers used for second scan only and genotyped in all animals).

A linkage map was constructed with 136 microsatellite markers including 116 markers used in the first genome scan of the MSU Duroc x Pietrain population [8] distributed across the 18 autosomes and 20 additional markers located on 9 chromosomes (SSC3 - 7, 12, 15, 16 and 18; 1 to 4 markers per chromosome; Additional file 1). All animals were genotyped for new markers, and 444 additional F_2 _animals not included in the first scan were also genotyped for 20 of the markers used in the first scan located on the 9 targeted chromosomes. The total genome length excluding the sex chromosomes was 3,089.6 Haldane cM with an average marker interval of 19.5 cM for the 9 chromosomes having additional markers and 28.2 cM for other chromosomes. The information content was increased by adding markers and animals (Figure 1).

Three least-squares Mendelian models for QTL analysis were fit to each trait for this study: a line-cross model, a half-sib model, and a combined line-cross and half-sib model, whereas only a line-cross model was applied to the first scan of this population which used 510 F_2 _pigs [8,9]. A total of 41 QTL were identified (Table 1). The line-cross analysis revealed 26 QTL, including 7 new QTL not detected in the first scan. The half-sib analysis revealed 12 QTL, and three additional QTL were detected using the combined line-cross and half-sib model. A total of 23 QTL were identified with the combined model, but 20 of these were already detected with either the line-cross or half-sib model. Thus, the combined model used in tandem with the line-cross and half-sib models facilitated identification of additional QTL not detected by either independent analysis. The significance threshold was determined by False Discovery Rate (FDR) and FDR was compared to conventional permutation tests for selected traits. A 5% FDR was more stringent than a 5% chromosome-wise level threshold and a 1% FDR was more stringent than a 5% genome-wise level threshold. For example, for 10^th ^rib backfat (BF10) at 19-wk of age the 5% FDR F-ratio of 6.79 was higher than the 5% chromosome-wise level threshold F-ratios of minimum 4.46 and maximum 5.69, and the 1% FDR F-ratio of 8.68 was higher than the 5% genome-wise level threshold F-ratio of 8.38.

**Table 1:** Position and significance level of growth trait QTL.

**Chr**^**1**^	**Position**^**2**^	Trait	**Type**^**3**^	**-log**_**10**_***P***^**4**^	**FDR**^**5**^	Flanking Markers	**Additive**^**6**^	**Dominance**^**7**^
4	25	22-wk total body fat tissue, kg	HS	3.17	0.0484	SW2509 - S0301		
	42	22-wk 10th-rib backfat, mm	HS	3.65	0.0209	S0301 - SW871		
	57	22-wk LM area, cm^2^	LC	3.29	0.0351	S0301 - SW871	-1.10 (0.29)	-0.66 (0.51)
	65	16-wk last-rib backfat, mm	HS	3.97	0.0120	S0301 - SW871		
6	25	22-wk empty body lipid, kg	HS	3.76	0.0179	S0099 - SW2406		
	26	22-wk total body fat tissue, kg	HS	3.29	0.0398	S0099 - SW2406		
	129	22-wk fat-free total lean, kg	LC	5.30	0.0006	S0220 - SW122	-0.35 (0.09)	0.44 (0.14)
	129	22-wk empty body protein, kg	LC	4.85	0.0016	S0220 - SW122	-0.13 (0.03)	0.14 (0.05)
	164	13-wk 10th-rib backfat, mm	LC	21.81	0.0000	SW1647 - SW1881	0.98 (0.10)	-0.74 (0.15)
	164	22-wk 10th-rib backfat, mm	LC	18.49	0.0000	SW1647 - SW1881	2.17 (0.25)	-1.37 (0.36)
	164	22-wk last-rib backfat, mm	LC	22.25	0.0000	SW1647 - SW1881	1.55 (0.17)	-1.24 (0.24)
	165	10-wk 10th-rib backfat, mm	LC	22.90	0.0000	SW1647 - SW1881	0.70 (0.07)	-0.57 (0.11)
	165	13-wk last-rib backfat, mm	LC	21.85	0.0000	SW1647 - SW1881	0.58 (0.06)	-0.39 (0.09)
	165	16-wk last-rib backfat, mm	LC	22.26	0.0000	SW1647 - SW1881	0.86 (0.10)	-0.81 (0.14)
	166	10-wk last-rib backfat, mm	LC	20.30	0.0000	SW1647 - SW1881	0.43 (0.05)	-0.30 (0.07)
	166	19-wk last-rib backfat, mm	LC	27.25	0.0000	SW1647 - SW1881	1.32 (0.13)	-1.03 (0.18)
	168	22-wk total body fat tissue, kg	LC	4.69	0.0023	SW1881 - SW322	0.57 (0.13)	-0.38 (0.19)
	169	19-wk 10th-rib backfat, mm	LC	25.29	0.0000	SW1881 - SW322	1.78 (0.18)	-1.61 (0.27)
	172	16-wk 10th-rib backfat, mm	LC	23.45	0.0000	SW1881 - SW322	1.38 (0.14)	-0.97 (0.22)
	174	22-wk empty body lipid, kg	LC	8.61	0.0000	SW1881 - SW322	0.46 (0.09)	-0.50 (0.14)
	229	22-wk fat-free total lean, kg	HS	3.38	0.0343	SW607 - SW2419		
7	44	10-wk LM area, cm^2^	HS	3.41	0.0325	S0064 - SW1369		
	48	19-wk LM area, cm^2^	HS	4.78	0.0024	S0064 - SW1369		
	138	19-wk LM area, cm^2†^	LC	5.02	0.0011	SW859 - SW2040	-1.38 (0.29)	-0.55 (0.62)
	169	10-wk LM area, cm^2†^	LC	3.51	0.0236	SW2040 - S0115	-0.43 (0.11)	0.05 (0.16)
	185	13-wk LM area, cm^2†^	LC	3.22	0.0391	S0115 - SW632	-0.49 (0.13)	0.26 (0.20)
8	137	22-wk empty body lipid, kg	HS	3.30	0.0397	S0017 - SW2160		
9	7	22-wk body weight, kg^†^	LC	3.10	0.0485	SW21 - SW983	-0.52 (0.71)	4.12 (1.10)
	9	22-wk empty body lipid, kg	CB	3.55	0.0433	SW21 - SW983		
	12	19-wk body weight, kg	CB	3.78	0.0297	SW21 - SW983		
11	91	19-wk last-rib backfat, mm	LC	3.98	0.0093	S0230 - SW66	0.20 (0.19)	-1.11 (0.27)
	93	19-wk 10th-rib backfat, mm	LC	3.37	0.0307	S0230 - SW66	-0.63 (0.28)	-1.28 (0.41)
	106	22-wk empty body lipid, kg	LC	3.28	0.0356	S0230 - SW66	-0.22 (0.15)	-1.03 (0.29)
15	39	16-wk LM area, cm^2†^	LC	3.19	0.0413	S0148 - SW1989	-0.75 (0.19)	0.05 (0.30)
	69	19-wk LM area, cm^2†^	LC	3.28	0.0356	S0088 - SW1683	-0.86 (0.22)	-0.01 (0.35)
	74	22-wk 10th-rib backfat, mm	HS	4.43	0.0048	SW1683 - SW906		
	96	19-wk 10th-rib backfat, mm	HS	4.38	0.0053	SW1983 - SW1119		
16	93	22-wk total body fat tissue, kg	LC	3.64	0.0186	SW2517	-0.47 (0.18)	-0.89 (0.31)
	93	19-wk 10th-rib backfat, mm	HS	3.36	0.0355	SW2517		
	93	22-wk body weight, kg	CB	3.57	0.0424	SW2517		
18	4	16-wk 10th-rib backfat, mm^†^	LC	3.54	0.0222	SW2540 - SW1023	0.55 (0.14)	0.23 (0.20)

^1^Chr = chromosome

^2^Position in Haldane cM

^3^LC = QTL declared as line-cross type; HS = half-sib type; CB = combined type.

^4^Negative logarithm of the comparison-wise p value of the test statistic against the null hypothesis of no QTL at the most likely position for the inferred QTL model.

^5^FDR = false discovery rate

^6^Estimates of additive effects with standard errors for LC QTL. The effects are expressed as (DD-PP)/2, where D = Duroc allele and P = Pietrain allele.

^7^Estimates of dominance effects with standard errors for LC QTL. The effects are expressed as DP-PD, where D = Duroc allele and P = Pietrain allele.

^†^New QTL detected with the line-cross model in the second scan.

**Figure 2:**
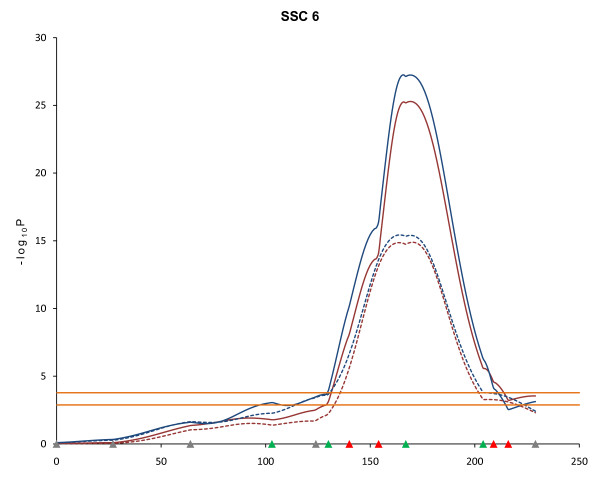
**Effect of additional markers and animals on statistical power for QTL detection**. Blue lines indicate last rib fat (LRF) QTL at 19-wk and red lines indicate 10^th ^rib backfat (BF10) QTL at 19-wk on SSC6. Solid lines are second scan results and dotted lines are first scan results. Marker positions are shown as triangles on the X-axis (gray, markers used for both QTL scans and genotyped only in 510 animals; green, markers used for both QTL scans and genotyped in all animals; red, markers used for second scan only and genotyped in all animals). Horizontal lines indicate significance thresholds (lower line, 5% FDR; upper line, 1% FDR).

Twelve highly significant QTL affecting fat deposition at different developmental stages were detected using the line-cross model on SSC6 between 164 and 174 cM (FDR ≤ 0.002), which is consistent with results of the first scan [8] (Table 1). The estimates of the additive effects of these QTL indicated that the Duroc alleles contributed to higher fat deposition (Table 1). For example, the QTL affecting BF10 at 22-wk of age had an estimated additive effect of 2.17 mm indicating that Duroc alleles contribute to larger measures of BF10. The addition of two markers into the *SW122 *- *SW18 *interval on SSC6, as well as the addition of more F_2 _pigs, narrowed the estimated QTL region and increased the statistical power. For last-rib backfat (LRF) at 19-wk of age, the 95% confidence interval decreased from 16 cM (160 cM - 176 cM) to 12 cM (163 cM - 175 cM) and the test statistic (-log_10_P) increased from 15.43 to 27.25 under the same model (Figure 2). Similarly for BF10 at 19-wk, the 95% confidence interval narrowed from 11 cM (163.5 cM - 174.5 cM) to 9.5 cM (164.5 - 174 cM) and the test statistic (-log_10_P) increased from 14.89 to 25.29 under the same model (Figure 2).

Results for SSC6 using the line-cross model also revealed significant QTL for 22-wk fat-free total lean tissue (FFTOLN) and 22-wk empty body protein (EBPRO) at 129 cM, consistent with results of the first scan [8]. In addition, three new QTL were discovered under the half-sib model. QTL for 22-wk empty body lipid (EBLIPID) and 22-wk total body fat tissue (TOTFAT) were detected at 25 and 26 cM, respectively, and a QTL for FFTOLN was detected at 229 cM.

**Figure 3:**
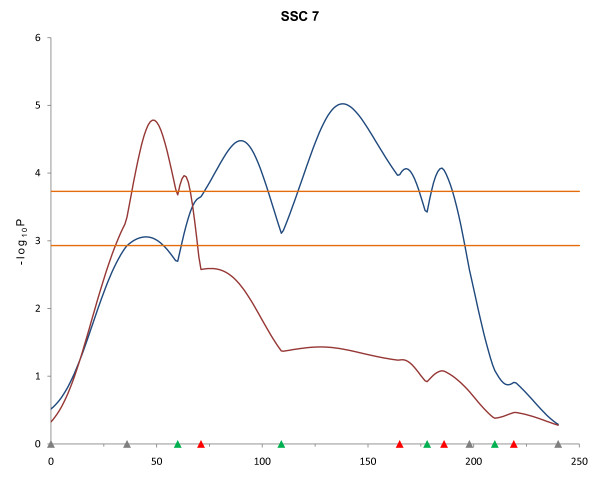
**QTL results determined by different models for *longissimus *muscle area (LMA) at 19 weeks of age on SSC7**. Blue and red lines indicate LMA QTL at 19-wk detected by line-cross and half-sib models, respectively (FDR ≤ 0.002). Marker positions are shown as triangles on the X-axis (gray, markers used for both QTL scans and genotyped only in 510 animals; green, markers used for both QTL scans and genotyped in all animals; red, markers used for second scan only and genotyped in all animals). Horizontal lines indicate significance thresholds (lower line, 5% FDR; upper line, 1% FDR).

Additional markers and F_2 _animals contributed to detection of new QTL using the line-cross model that were not detected in the first scan on SSC7, 15 and 18. In addition, QTL for fat traits detected in the first scan on SSC11 and SSC16 were confirmed in the second scan. For SSC7, QTL were detected for *longissimus *muscle area (LMA) at 19-wk of age (FDR < 0.002) and for LMA at 10- and 13-wk (FDR < 0.04). Half-sib analysis of SSC7 revealed QTL for LMA at 10- and 19-wk of age in the *S0064 *- *SW1369 *interval that differed in location by 125 cM and 90 cM, respectively (Table 1). QTL for LMA at 19-wk were detected by both line-cross and half-sib analyses and were significant at the 1% FDR level, but their locations were in completely different positions (Figure 3).

**Figure 4:**
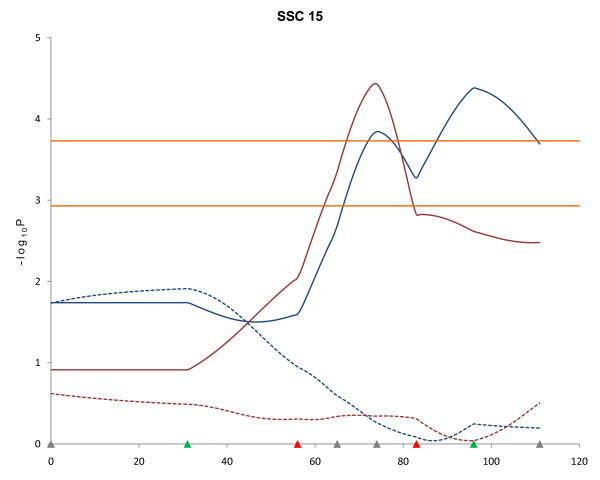
**QTL results determined by different models for 10**^**th **^**rib backfat (BF10) at 19 and 22 weeks of age on SSC15**. Blue and red solid lines indicate BF10 at 19- and 22-wk, respectively, detected with the half-sib model (FDR ≤ 0.005). Blue and red dotted lines indicate BF10 at 19- and 22-wk, respectively, detected with the line-cross model. Marker positions are shown as triangles on the X-axis (gray, markers used for both QTL scans and genotyped only in 510 animals; green, markers used for both QTL scans and genotyped in all animals; red, markers used for second scan only and genotyped in all animals). Horizontal lines indicate significance thresholds (lower line, 5% FDR; upper line, 1% FDR).

No QTL for growth traits were observed on SSC15 in the first QTL scan of this population [8]. However, the second scan of this chromosome using the line-cross model revealed QTL at 39 and 69 cM for LMA at 16- and 19-wk, respectively (FDR ≤ 0.04, Table 1). Using the half-sib model for SSC15, significant QTL were detected for BF10 at 19- and 22-wk at 96 and 74 cM, respectively (FDR ≤ 0.005). However, the line-cross analysis did not detect significant BF10 QTL in this SSC15 region (Figure 4).

A QTL for body weight at 22-wk was detected using the line-cross model in the *SW21 *- *SW983 *marker interval at the proximal end of SSC9 (FDR ≤ 0.05). QTL for body weight at 19-wk and EBLIPID were also detected in this same chromosomal region using the combined model (FDR ≤ 0.05). A new QTL for BF10 at 16-wk was mapped by line-cross analysis at 4 cM between markers *SW2540 *and *SW1023 *on SSC18. The SW1808 and SW2540 markers were added to SSC18 proximal to *SW1023*, thus extending the SSC18 map and facilitating further QTL detection on this chromosome.

Analysis of SSC4 using the line-cross model confirmed a QTL at 57 cM for 22-wk LMA that was observed in the first scan [8]. This QTL had an estimated additive effect of -1.1 mm^2 ^of LMA indicating that Pietrain alleles contribute to a larger LMA. Significant SSC4 QTL were identified using the half-sib model for BF10 at 22-wk and LRF at 16-wk in the *S0301 *- *SW871 *region and also for TOTFAT at 25 cM.

New QTL were also detected using the half-sib model on SSC8 for EBLIPID at 137 cM (FDR ≤ 0.04) and on SSC16 for 19-wk BF10 at 93 cM (FDR ≤ 0.04). This position on SSC16 also included a QTL for TOTFAT detected using the line-cross analysis and a QTL for body weight at 22-wk detected using the combined analysis.

## Discussion

Increasing the number of markers and animals for the genome scan of our Duroc x Pietrain resource population facilitated detection of new QTL (SSC7, 15 and 18), as well as confirmation of previously identified QTL affecting growth traits (SSC4, 6, and 16) using the line-cross analysis [8]. A QTL for LMA at 22-wk was confirmed on SSC4. On SSC6, 14 QTL affecting fat deposition traits were confirmed, although three QTL for LMA identified in the first scan were not significant in the second scan. On SSC7, three new QTL for LMA were identified, whereas a QTL for ADG located in the *S0115 *- *SWR773 *interval in the first scan was not detected. Two new QTL for LMA were detected on SSC15. Six QTL on SSC16 had been detected in the first scan but only one QTL for TOTFAT was confirmed in the second scan. Many of these QTL which were significant at the 5% chromosome-wise level in the first scan were not detected in the second scan, either because the 5% FDR threshold used for the second scan was slightly more stringent than the 5% chromosome-wise level threshold or because these were false positives in the first scan.

A QTL for LMA at 22-wk detected with the line-cross analysis, and QTL for LRF at 16-wk and BF10 at 22-wk detected with the half-sib analysis were localized in the *S0301 *- *SW871 *interval on SSC4. A possible explanation for these results could be QTL alleles of Pietrain origin affecting LMA, and QTL alleles influencing backfat thickness segregating in each founder breed. A QTL affecting fatness in this interval has been confirmed by many previous studies [10-13], including a report by Andersson et al. [13] of the first pig QTL for growth and fatness on SSC4 in a Wild boar x Large White cross. Also, Cepica et al. [10] reported a QTL for LMA in this same region and demonstrated that Pietrain alleles were associated with increased meat and decreased fat content in a Wild boar x Pietrain cross.

Strong evidence for QTL affecting fatness was revealed at marker interval *SW1647 *- *SW1881 *on SSC6. QTL for BF10 and LRF at different stages of growth (measured by ultrasound) were highly significant (FDR < 0.001). The estimates of the additive effects suggest that Duroc alleles contributed to larger measures of BF10 and LRF. However the estimates of the dominance effects for these QTL were negative. In the first scan, the predominant location for most of the BF10 and LRF QTL was estimated to be distal to marker *SW1881*. However, with the addition of marker *SW1647*, these QTL were determined to be located in the interval *SW1647 *- *SW1881 *in the second scan. This region includes the *leptin receptor *(*LEPR*) gene located on SSC6q3.3-3.5 [14] and Óvilo et al. [15] reported a QTL for fatness in this same region. Muñoz et al. [16] reported the effect of QTL and *LEPR *alleles in this region to be significant for backfat thickness and also for body weight. Our results are in agreement with those of Muñoz et al. [16] regarding backfat QTL. However, we did not find evidence for a QTL affecting body weight on SSC6, although body weight was positively correlated with BF10 and LRF in our population.

Three other regions on SSC6 included QTL for body composition traits determined by either the line-cross analysis or the half-sib analysis. In the line-cross analysis, QTL for FFTOLN and EBPRO at 22-wk mapped to 129 cM and this result is in agreement with a study reported by Mohrmann et al. [17]. Two additional QTL regions affecting EBLIPID and TOTFAT, and FFTOLN were detected by the half-sib analysis and were located at the proximal and distal ends of SSC6, respectively.

QTL for LMA at 10- and 19-wk of age were detected using both the line-cross and the half-sib models on different regions of SSC7. A newly detected QTL for LMA at 19-wk under the line-cross model was mapped to 138 cM with the contribution of the Pietrain allele increasing LMA, whereas the half-sib model revealed a QTL for LMA at 19-wk at 48 cM. Nagamine et al. [18] reported an LMA QTL in a Large White population that spanned most of SSC7 and Uemoto et al. [19] reported an LMA QTL segregating in a Duroc population with a relative peak location in between the QTL detected using the line-cross and half-sib models in the current study.

QTL for LMA located on SSC15 were also identified in the second scan. The QTL for LMA at 19-wk was detected in the *S0088 *- *SW1683 *interval in a region that includes *MSTN *(*myostatin*) which is considered to be a candidate gene for muscle hypertrophy [20]. Stinckens et al. [21] reported a polymorphism in the porcine *MSTN *promoter region MEF3 binding site, which could potentially abolish enhancer activity, and that had a very high allele frequency in the Pietrain breed. Thus, its effect could be associated with the higher muscularity of the Pietrain breed. Two QTL influencing backfat thickness were identified on SSC15 using the half-sib analysis. A QTL for BF10 at 22-wk was located in the *SW1683 *- *SW906 *interval and a QTL for BF10 at 19-wk was located in the *SW1983 *- *SW1119 *interval. This latter region is consistent with a QTL for 1st rib fatness detected in a four-way cross by Harmegnies et al. [22].

The marker *SW2517 *located on SSC16q2.2 has been reported to be linked to a QTL affecting fatness at later stages of growth [23]. In the first scan, five QTL (body weight and BF10 at 19-wk, body weight and LRF at 22-wk, and TOTFAT) were detected near *SW2517*. A QTL for ADG was also identified on SSC16 distal to this region. In the second scan, QTL for TOTFAT detected with the line-cross analysis, BF10 at 19-wk detected with the half-sib analysis and body weight at 22-wk detected with the combined analysis were mapped to 93 cM at marker *SW2517*. In addition, suggestive QTL for EBLIPID (FDR < 0.06), TOTFAT (FDR < 0.066) and Age at 105 kg (FDR < 0.073) under the combined model were located at the same position. No additional *SW2517 *animal genotypes were included in the second scan, although two new markers flanking *SW2517 *were genotyped across the full population. For marker *SW2517*, three *SW2517 *alleles were segregating and the number of phase known informative meioses was 385 of 510 animals. The allele associated with fatness and heavier body weight originated only from the Pietrain, whereas the other two alleles were segregating in both the Pietrain and Duroc founder breeds. Thus segregation patterns of alleles at this marker allowed detection of QTL by all three models. Liu et al. [24,25] reported that a QTL influencing backfat thickness using both line-cross and combined analyses was located in the same SSC16 region for their Duroc x Pietrain population. The *prolactin receptor *(*PRLR*) gene located in this region [26] is well-known as a candidate gene affecting reproductive traits in pigs [27-30]. Prolactin also stimulates fat deposition and weight gain, and stimulates increases in white adipose tissue leptin mRNA and plasma leptin levels [31,32]. Freemark et al. [33] provided evidence that the absence of *PRLR *in knockout mice was accompanied by reduced body weight gain after 16 weeks of age and decreased abdominal fat mass. Recently, Lu et al. [34] demonstrated that polymorphisms in the *PRLR *gene were associated with growth traits in cattle. Based on results for other species and our localization of QTL on SSC16, *PRLR *may be a candidate gene for growth and fat deposition in pigs and further research is warranted.

A QTL for BF10 at 16-wk was identified at the proximal end of SSC18. Malek et al. [35] detected QTL for backfat thickness in the same SSC18 region in a Berkshire x Yorkshire population. A novel QTL for body weight at 22-wk was also identified on SSC9 in the second scan. This QTL had a dominance effect of 4.12 kg, which indicated that the heterozygous genotype contributed to heavier body weight at 22-wk. A QTL for body weight at 19-wk detected by the combined analysis was located in the same interval. A QTL for body weight at 3-wk was reported in the same SSC9 region [24], but no other body weight QTL have been reported in the *SW21 *- *SW983 *interval.

## Conclusions

Additional markers and animal genotypes contributed to refine QTL positions and increase the statistical power. The application of different QTL analysis models made it possible to detect new QTL segregating either between or within breeds. In total, 26 QTL with the line-cross model, 12 QTL with the half-sib model and 3 additional QTL with the combined line-cross and half-sib model were detected for pig growth traits. Analysis using the line-cross model was most powerful for detecting QTL, whereas the combined model which assumed QTL to be segregating at different allelic frequencies in the founder populations was less powerful than the line-cross or half-sib models. This result was not unexpected because the population was designed to exploit between breed differences and markers were selected in such a way that they were more informative to declare breed of origin QTL than for detecting QTL using the half-sib analysis. However, our analysis shows that there is substantial segregation within breed that can be tracked (although to a lesser extent) by using the sire haplotype probabilities either alone (half-sib analysis) or jointly with the breed origin probabilities (combined analysis).

## Methods

### Animals and phenotypes

Animals from a three-generation Duroc x Pietrain resource population developed at Michigan State University and described by Edwards et al. [8] were used for this study. Animal protocols were approved by the Michigan State University All University Committee on Animal Use and Care (AUF# 09/03-114-00). The population was established from 4 F_0 _Duroc sires and 15 F_0 _Pietrain dams. The F_2 _pigs were produced from 50 F_1 _females and 6 F_1 _males, and were born in 141 litters across 11 farrowing groups. The second genome scan for this study included the 510 F_2 _animals used in the first genome scan along with an additional 444 F_2 _animals. The 954 total pigs included all F_2 _animals from this population for which complete growth phenotypes are available. Descriptive statistics for phenotypes used in this study are presented in Table 2.

**Table 2:** Number of records, means, and SD for growth traits measured.

Trait	N	Mean	SD
Birth weight (kg)	954	1.53	0.32
3-wk weight (kg)	954	5.69	1.48
6-wk weight (kg)	953	12.04	2.84
10-wk weight (kg)	954	26.43	4.84
10-wk 10th-rib backfat (mm)^1^	954	7.96	1.77
10-wk *longissimus *muscle area (cm^2^) ^1^	954	11.55	2.54
10-wk last-rib backfat (mm) ^1^	954	6.11	1.06
13-wk weight (kg)	954	41.66	6.60
13-wk 10th-rib backfat (mm) ^1^	954	9.74	2.68
13-wk *longissimus *muscle area (cm^2^) ^1^	954	16.98	3.35
13-wk last-rib backfat (mm) ^1^	954	7.13	1.38
16-wk weight (kg)	954	62.28	8.27
16-wk 10th-rib backfat (mm) ^1^	954	12.35	3.44
16-wk *longissimus *muscle area (cm^2^) ^1^	954	24.85	3.82
16-wk last-rib backfat (mm) ^1^	954	9.57	2.28
19-wk weight (kg)	954	80.79	9.84
19-wk 10th-rib backfat (mm) ^1^	954	15.90	5.02
19-wk *longissimus *muscle area (cm^2^) ^1^	954	31.39	4.19
19-wk last-rib backfat (mm) ^1^	954	11.79	3.29
22-wk weight (kg)	954	100.05	10.87
22-wk 10th-rib backfat (mm) ^1^	954	19.89	6.40
22-wk *longissimus *muscle area (cm^2^) ^1^	954	37.09	4.83
22-wk last-rib backfat (mm) ^1^	954	14.35	4.16
10 - 22 wk ADG (g/d)	954	878.04	105.42
Age at 105 (kg) ^2^	954	157.42	13.64
22-wk total body fat tissue (kg) ^3^	954	24.94	6.96
22-wk fat-free total lean tissue (kg) ^3^	954	38.35	4.45
22-wk empty body protein (kg) ^3^	954	15.01	1.67
22-wk empty body lipid (kg) ^3^	954	21.96	4.23

^1^10th rib backfat, last rib backfat, and *longissimus *muscle area at 10, 13, 16, 19, and 22 wk of age estimated using B-mode ultrasound (Pie Medical 200SLC, Classic Medical Supply Inc., Tequesta, FL).

^2^Age at 105 kg calculated following National Swine Improvement Federation guidelines [40].

^3^Total body fat tissue, fat-free total lean tissue, empty body protein, and empty body lipid at 22 wk of age calculated by using equations similar to those used by Wagner et al. [41].

### Markers and genotyping

Based on the first genome scan results using 124 markers [8,9], 9 chromosomes (SSC3, 4, 5, 6, 7, 12, 15, 16 and 18) with significant QTL were selected for additional marker genotyping. Twenty new microsatellite markers were selected from the publicly available pig genome linkage map http://www.marc.usda.gov/genome/swine/swine.html that map within the QTL regions on these chromosomes (Additional file 1). New markers were confirmed to be informative in the MSU population by genotyping of F_0 _pigs. All F_0_, F_1_, and the 954 F_2 _pigs were genotyped for the 20 new makers, and the 444 additional F_2 _pigs were also genotyped for 20 markers flanking the QTL regions on the 9 selected chromosomes. Genotyping was performed at a commercial laboratory (GeneSeek Inc., Lincoln, NE). Sex-averaged genetic linkage maps were constructed using CRI-MAP version 2.4 [36] and converted to the Haldane map function [37].

### Statistical analysis

QTL mapping was performed using least-squares regression with line-cross, half-sib and combined line-cross and half-sib models. Genome-wise significance thresholds were determined by false discovery rate (FDR) [38]. QTL detected using the line-cross or half-sib model were declared using a FDR threshold of 5%, and then additional QTL detected with the combined line-cross and half-sib model were declared when such QTL had not previously been detected by either the line-cross or half-sib model.

Under the line-cross model it is assumed that the two founder lines are fixed for alternative alleles at the QTL affecting the traits of interest [1]. The QTL Express software [39] was used to estimate the probability of each F_2 _individual being homozygous for two Duroc alleles (P_11_), homozygous for two Pietrain alleles (P_22_), or heterozygous (P_12 _or P_21_) at fixed 1-cM intervals across the genome. By denoting the effect of P_11 _as positive additive (a), the effect of P_12 _+ P_21 _as dominance (d) and the effect of P_22 _as negative additive (-a), the following linear model was fitted at every cM across the genome.

$$y_{j}=X_{j}b+aPa_{j}+dPd_{j}+e_{j}$$

Where *y*_*j *_is the phenotype of F_2 _progeny *j*, *X*_*j *_and *b *are the design matrix and solution vector for the fixed effects, respectively, *a *and *d *are the estimated additive and dominance effects of a putative QTL at the given location, respectively, *Pa*_*j *_is the conditional probability of animal *j *to carry two Duroc alleles, *Pd*_*j *_is the conditional probability of animal *j *to be heterozygous, and *e*_*j *_is the residual error. The model for all traits included sex of animal and litter as fixed effects and the model for 10- to 22-wk ADG included 10-wk body weight as a covariate.

For the half-sib analysis, the F_2 _individuals were treated as 6 paternal half-sib families which assumes no fixation of the QTL alleles in the founder lines. QTL Express [39] was used to calculate the probabilities of individuals of allele (P_1_) or allele (P_2_) from the common Duroc parent (P_12 _or P_21_) [5]. In these analyses contrasts were made between the two haplotypes of every F_1 _sire.

$$y_{ij}=X_{ij}b+s_{i}+\alpha {\text{HS}}_{i}Ps_{ij}+e_{ij}$$

Where *y*_*ij *_is the phenotype of F_2 _progeny *j *of F_1 _sire *i*, *X*_*ij *_and *b *are the design matrix and the solution vector for fixed effects, respectively, *s*_*i *_is the effect of the *i*th F_1 _sire, *α*HS_i _is the substitution effect for the two putative QTL alleles (P_1 _or P_2_) carried by the *i*th F_1 _sire, *Ps*_*ij *_is the probability that the F_2 _individuals inherited the arbitrary allele (P_1_) from F_1 _sire *i*, and *e*_*ij *_is the residual error.

The combined line-cross and half-sib model included,

$$y_{ij}=X_{ij}b+s_{i}+aPa_{ij}+dPd_{ij}+\alpha CB_{i}Ps_{ij}+e_{ij}$$

Where *y*_*ij *_is the phenotype of F_2 _progeny *j *of F_1 _sire *i*, *X*_*ij *_and *b *are the design matrix and the solution vector for fixed effects, respectively, *s*_*i *_is the effect of the *i*th F_1 _sire, *a *and *d *are the additive and dominance effects of breed-origin alleles, respectively, *Pa*_*ij *_and *Pd*_*ij *_are the corresponding breed-origin coefficients, *αCB*_*i *_is the substitution effect for the two putative QTL alleles carried by the *i*th F_1 _sire, *Ps*_*ij *_is the probability that the F_2 _individuals inherited the arbitrary allele (P_1_) from F_1 _sire *i*, and *e*_*ij *_is the residual error. In this model, *a *and *d *account for the average effects of breed origin alleles through both the F_1 _sire and the F_1 _dam and *αCB*_*i *_represents the difference between the two QTL alleles that a given F_1 _sire received from the two parental breeds as a deviation from their average additive effect [6].

## Authors' contributions

IC carried out the data analyses and drafted the manuscript. JPS participated in design of the study and supervised the data analyses. ROB coordinated development of the resource population and participated in design of the study. NER prepared the DNA samples and identified segregating markers. JMR contributed to design of the study. CWE coordinated the project, participated in design of the study and helped draft the manuscript. All authors read and approved the final manuscript.

## Authors' information

JMR is currently Senior Scientist, Pfizer Animal Genetics, Kalamazoo, MI.

## Supplementary Material

Additional file 1 **Genetic maps constructed for the first and second genome scans of the Michigan State University Duroc x Pietrain resource population**. All markers used for the first and second genome scans are listed and genetic maps are shown for both scans. Click here for file
