# Supplementary material for: Application of alternative models to identify QTL for growth traits in an F2 Duroc x Pietrain pig resource population
Source: BMC Genet. 2010 Nov 1;11:97. doi: 10.1186/1471-2156-11-97 (PMC2989936; doi:10.1186/1471-2156-11-97)
Supplement: Additional file 1 — Genetic maps constructed for the first and second genome scans of the Michigan State University Duroc x Pietrain resource population. All markers used for the first and second genome scans are listed and genetic maps are shown for both scans. [file 1471-2156-11-97-S1.PDF]

Additional file 1. Genetic maps constructed for the first and second genome scans of the Michigan State University Duroc x Pietrain resource population.

Chr[1]	Marker	1st scan[2] Kosambi cM	2nd scan Haldane cM
1	SW1514	0.0	0.0
	SW1515	21.1	25.4
	S0008	49.7	61.8
	S0331	77.5	97.0
	SW974	108.9	137.6
	S0056	179.7	247.4
	SW1301	235.1	328.7
2	SWR2516	0.0	0.0
	SW240	41.0	56.2
	S0170	53.7	70.5
	SW1026	64.8	82.8
	S0370	93.4	119.2
	SW1844	103.2	129.9
	S0378	110.1	137.3
	S0036	142.0	178.8
3	SW274	0.0	0.0
	SW2021	22.7	27.7
	S0206[‡]	68.7	94.8
	SWR978[†]		112.9
	ACTG2	86.4	116.5
	SW2141[†]		127.1
	SW2047[‡]	101.3	134.8
	SW2408	124.3	163.4
	S0002	132.4	174.1
	SW1327	141.2	182.8
	SW2532	159.6	204.5
4	SW2404[‡]	0.0	0.0
	SW2509[†]		10.0
	S0301[‡]	29.2	35.3
	SW871	54.1	66.3
	SW2454	61.8	74.6
	S0107	73.8	88.2
	S0214	88.0	104.2
	S0097	131.3	164.5
5	SW413	0.0	0
	ACR	11.5	12.8
	SWR453	53.4	70.5
	SW2	81.5	106
	S0005	108.9	140.4
	S0018	127.4	162.8
	IGF1[†]		179.1
	SW995	147.6	185.5
	SW378	159.7	199.6

Chr	Marker	1st scan Kosambi cM	2nd scan Haldane cM
6	S0099	0.0	0.0
	SW2406	22.4	27.3
	SW2525	50.7	64.2
	S0087[‡]	81.4	103.4
	S0220	98.2	123.6
	SW122[‡]	103.9	129.5
	SW2173[†]		140.0
	SW1647[†]		153.7
	SW1881[‡]	135.8	167.0
	SW322[‡]	164.8	204.1
	SW1328[†]		208.9
	SW607[†]		216.1
	SW2419	181.1	229.6
7	S0025	0.0	0.0
	S0064	28.1	35.6
	SW1369[‡]	48.0	59.8
	SW2019[†]		70.7
	SW859[‡]	92.5	109.2
	SW2040[†]		164.5
	S0115[‡]	135.8	177.7
	SW632[†]		185.5
	SWR773	151.6	197.7
	S0101[‡]	164.0	209.7
	S0212[†]		219.4
	SW764	186.7	240.7
8	SW2410	0.0	0.0
	SW905	22.9	28.0
	SWR1101	55.0	69.7
	S0017	95.1	124.4
	SW2160	110.7	142.4
	SW1085	124.4	158.0
	S0178	165.5	214.4
9	SW21	0.0	0.0
	SW983	13.2	14.9
	SW911	43.1	53.3
	SW2401	63.7	78.0
	SW539	72.4	87.5
	SW989	97.0	117.9
	SW2116	127.4	157.0
10	SWR136	0.0	0.0
	SW249	20.2	24.2
	SWC19	44.3	53.9
	SW1041	56.8	67.9
	SW920	79.4	95.5

Additional file 1 (Continued)

Chr[1]	Marker	1st scan Kosambi cM	2nd scan Haldane cM
11	S0391	0.0	0.0
	S0071	53.0	77.0
	S0230	65.2	90.7
	SW66	119.0	169.1
12	SW2490	0.0	0
	SW957‡	31.2	40.4
	SW874	47.2	59
	SW37†		64.8
	S0090	61.0	76.2
	SWC23†		97.5
	SW2180‡	94.1	117.4
13	S0219	0.0	0.0
	SWR1941	13.7	15.6
	SW344	40.9	49.8
	SWR1008	54.2	64.9
	S0068	65.1	76.9
	SW398	85.8	101.8
	SW2440	103.2	122.2
	S0215	122.6	145.3
14	SW857	0.0	0.0
	SW510	26.6	33.4
	SW210	45.9	56.3
	SW886	64.7	78.6
	SW55	85.2	103.1
	SW1557	95.6	114.6
	SWC27	117.6	141.3

Chr	Marker	1st scan Kosambi cM	2nd scan Haldane cM
15	SW1204	0.0	0.0
	S0148‡	24.7	31.2
	SW1989†		56.3
	S0088	54.8	64.5
	SW1683	64.5	73.6
	SW906†		82.8
	SW1983‡	82.2	95.9
	SW1119	96.0	111.4
16	S0111	0.0	0.0
	SW419‡	30.9	39.4
	SW1454†		66.0
	SW2517	64.8	93.0
	SW1897†		127.7
	S0061‡	99.5	143.0
17	SWR1004	0.0	0.0
	SW2441	22.7	27.7
	SW1031	42.9	51.9
	SW2427	94.9	127.1
18	SW1808†		0.0
	SW2540†		3.2
	SW1023‡	0.0	37.9
	SW1984‡	43.1	96.6
	S0062	54.9	109.6

[1]Chr = chromosome

[2]First scan reported in Edwards et al. [8]

†Additional makers used for the second scan

‡Flanking markers used for the second scan
